# Body mass index and blood glucose in psychiatric and general practice populations

**DOI:** 10.1192/pb.bp.115.051219

**Published:** 2016-06

**Authors:** Sarah McAvoy, Matthew Cordiner, Jackie Kelly, Laura Chiwanda, Christine Jefferies, Kirsteen Miller, Polash Shajahan

**Affiliations:** 1 NHS Lanarkshire, Scotland, UK; 2 University of Glasgow, Scotland, UK

## Abstract

**Aims and method** Using a retrospective observational approach, we aimed to discern whether there was a difference in metabolic parameters between psychiatric and general practice populations in the same locality. Second, we aimed to establish differences in metabolic parameters of patients taking olanzapine, clozapine or aripiprazole.

**Results** Patients with psychiatric illness had a body mass index (BMI) comparable to that of the general practice population (28.7 *v*. 29.7 kg/m^2^), but blood glucose was significantly lower in the general practice population (4.8 *v*. 6.1 mmol/L). Olanzapine was associated with the lowest BMI (26.1 kg/m^2^) and aripiprazole the highest (32.2 kg/m^2^), with no difference in blood glucose between antipsychotics.

**Clinical implications** Awareness of environmental factors and how they affect individuals is important and medications are not the only cause of metabolic effects. There may be a channelling bias present, meaning practitioners are cognisant of potential metabolic effects prior to prescribing. Overall monitoring of physical health is important regardless of potential cause.

Patients with severe and enduring mental illness experience excess mortality, which appears to be linked to socioeconomic deprivation.^1^ Increased cardiovascular mortality is seen in such patient groups and recording of cardiovascular risk factors occurs at an earlier age.^2^ With regard to risk factors for cardiovascular disease, our accepted knowledge has become that the oral antipsychotics olanzapine^3,4^ and clozapine^5,6^ are most likely to be associated with weight gain, thereby high body mass index (BMI), potential diabetes mellitus and adverse cardiovascular outcomes. Indeed, their propensity for causing weight gain may correlate positively with antipsychotic effectiveness.^7^ The second-generation antipsychotic aripiprazole has been considered to cause less weight gain than for example clozapine or olanzapine. Large head-to-head Cochrane reviews support differences in such comparative weight gain favouring aripiprazole in randomised controlled trials.^8^ However, the authors of this Cochrane review questioned whether this translates to similar findings in clinical practice.

In clinical practice, antipsychotic medications are prescribed for various mental disorders. Clinicians are informed about likely beneficial and adverse effects of different antipsychotics by a number of sources, such as peer-reviewed journals, attended symposia and, perhaps more controversially, directly by pharmaceutical companies. The last does give rise to potential bias in data presentation and interpretation.

## Method

We aimed to identify psychiatric patient cohorts in our locality. This was with a view to examining the three antipsychotics of interest which are implicated in weight change and blood glucose regulation: olanzapine, clozapine and aripiprazole. We examined routine health measures including BMI, blood glucose and other parameters detailed below. The psychiatric populations identified were those with severe and enduring mental illness (*n* = 46), acute admissions to general psychiatry (*n* = 36) and admissions to the psychiatric intensive care unit (PICU) (*n* = 39). The total of the psychiatric population (*n* = 121) was compared with individuals from two local general practices who had presented for a routine physical health check-up (*n* = 80). The general practice population were identified as non-diabetic and not on antipsychotic medication. The project was registered with our clinical quality department.

Patients with severe and enduring mental illness were invited by letter to attend a physical health screening clinic. An information sheet which explained the importance of such an intervention was also sent out. Written consent was obtained from patients at the first meeting and it was explained that this new initiative was not designed to replace the annual general practice screening but instead to supplement it. The clinic was run by registered mental health nurses and trained clinical support workers. The rationale behind choosing the specific groups of patients was to examine a mix of patients seen routinely within clinical psychiatry. Those with severe and enduring mental illness were considered most likely to have the longest duration of contact with psychiatric services and theoretically greatest lifetime exposure to antipsychotic medication. Those from the acute admissions unit and PICU may have had less exposure to antipsychotic medication. The general practice group would serve as a comparison with no recorded exposure to antipsychotics.

We examined the link between the use of antipsychotics with perceived different propensities for weight gain (olanzapine, clozapine and aripiprazole) and associations with BMI and blood glucose levels. Long-acting antipsychotic injections were also analysed as a comparator. We also scrutinised duration of exposure to the antipsychotic in question and also any antipsychotic prescribed beforehand. This was to discern whether the antipsychotic of interest had been prescribed for a relatively short period of time and whether the previously prescribed antipsychotic may have influenced BMI and blood glucose changes. Our primary null hypothesis was that in clinical practice, BMI and blood glucose levels for the psychiatric population would not differ significantly from the local general practice population. Our secondary null hypothesis was that in clinical practice there would be no differences in BMI or blood glucose between the antipsychotics of interest. For nominal data one-way analysis of variance (ANOVA) and corresponding two-tailed *t*-tests were performed. For categorical data the χ^2^ or Fisher exact statistic was used.

## Results

Table 1 shows clinical, physical and lifestyle measures for the psychiatric and general practice populations. The psychiatric population had a greater proportion of men (*P* = 0.03) and was significantly older than the general practice population (t(173) = 7.5, *P*<0.0001). The BMI of psychiatric and general practice patients did not differ significantly, but was 1 kg/m^2^ lower in the psychiatric population. The general practice cohort had significantly lower blood glucose measures than psychiatric patients (t(130) = 5.1, *P*<0.0001). Mean blood pressure was within acceptable limits, but 9.1% of the psychiatric patients and 20% of the general practice population had a diastolic pressure equal to or greater than 90 mmHg.

**Table 1:** Clinical, metabolic and lifestyle measures

	All psychiatric patients (*n* = 121)	General practice (*n* = 80)	*P*
Male, *n* (%)	72 (60)	35 (44)	0.03

Mean age, years (95% CI)	37.8 (35.2–40.3)	47.4(47.0–47.8)	<0.001

Contact with mental health services, *n* (%)			
<1 year	16 (13.2)		
1–3 years	17 (14.0)		
>3 years	89 (73.6)		
>10 years	62 (51.2)		

Primary ICD-10 diagnosis, *n* (%)			
Bipolar affective disorder	22 (18.2)		
Schizophrenia/psychotic disorders	63 (52.1)		
Depressive disorder with or without psychosis	13 (10.7)		
EUPD/other personality disorder^a^	9 (9.1)		
Acute stress reaction	6 (5.0)		
Other^b^	8 (6.7)		

BMI, kg/m^2^			
Mean (95% CI)	28.7 (27.5–30.0)	29.7 (29.3–30.2)	ns
Range	18–46	17–55	
<25, *n/N* (%)	25/95 (26.3)	18 (22.5)	
25–30 (overweight), *n/N* (%)	32/95 (33.7)	26 (32.5)	
30–40 (obese), *n/N* (%)	33/95 (34.7)	29 (36.3)	
40+ (very obese), *n/N* (%)	4/95 (4.2)	7 (8.8)	

Blood glucose,^b^ mmol/L			
Mean (95% CI)	6.1 (5.6–6.5)	4.8 (4.8–4.9)	<0.001
⩾5.5, *n/N* (%)	57/93 (61.3)	18 (23)	
⩾7.0, *n/N* (%)	31/93 (33.3)	1 (1.25)	
⩾11.0, *n/N* (%)	6/93 (6.5)	0 (0)	

Blood pressure			
Mean systolic (95% CI), mmHg	126 (124–130)	126 (125–127)	
Mean diastolic (95% CI), mmHg	79 (77–81)	80 (76–83)	
Systolic ⩾160 mmHg, *n* (%)	2 (1.7)	1 (1.25)	
Diastolic ⩾90 mmHg, *n* (%)	11 (9.1)	8 (20)	

Regular cigarette smoking, *n/N* (%)	77/120 (64)	34 (43)	0.004

Admit to drinking ⩾21 alcohol units/week, *n* (%)	24 (19.8)	9 (11)	ns

BMI, body mass index; EUPD, emotionally unstable personality disorder; ns, not significant.

a. EUPD *n* = 7, dissocial personality disorder *n* = 2.

b. Opiate dependency *n* = 1, unclear diagnosis *n* = 7.

Table 2 describes the pattern of antipsychotic prescription across the different psychiatric cohorts. In terms of our antipsychotics of interest, olanzapine was used in 15.7%, clozapine in 9% and aripiprazole in 11.6% of all psychiatric patient groups combined. Median and range of lifetime cumulative daily dosage for the specific antipsychotics were as follows: aripiprazole 14.4 mg/day (5.0–31.3), olanzapine 16.6 mg/day (5.0–22.2) and clozapine 390 mg/day (250–470). Antipsychotic polypharmacy occurred in three patients on olanzapine, two patients on aripiprazole and one patient on clozapine.

**Table 2:** Antipsychotic prescribing patterns in psychiatric subpopulations

	All psychiatric populations (*n* = 121)	Severe and enduring mental illness (*n* = 46)	Acute admissions unit (*n* = 36)	PICU (*n* = 39)
Prescribed at time of admission, *n*				
Amisulpride	2	0	0	2
Aripiprazole	14	7	3	4
Chlorpromazine	1	0	0	1
Clozapine	12	6	3	3
Flupentixol LAI	3	1	0	2
Fluphenazine LAI	1	1	0	0
Haloperidol	2^b^	1^d^	1	0
Olanzapine	19	9	4	6
Paliperidone	5	2	2	1
Pipothiazine LAI	1	0	1	0
Quetiapine	20^a^	7	8^c^	5
Risperidone	10^b^	5^d^	2	3
Sulpiride	1	0	0	1
Zuclopenthixol LAI	16	8	5	3
None	16	0	8	8

Combination antipsychotics, *n* (%)	8 (6.6)	3 (6.5)	0 (0)	2 (5.1)

Percentage on antipsychotics	86.8	100	77.8	79.5

LAI, long-acting injection; PICU, psychiatric intensive care unit.

a. Quetiapine combined wiht flupentixol *n* = 1, pipothiazine *n* = 1.

b. Risperidone combined with haloperidol *n* = 1.

c. Quetiapine combined with pipothiazine *n* = 1.

d. Haloperidol combined with risperidone *n* = 1.

**Fig. 1:**
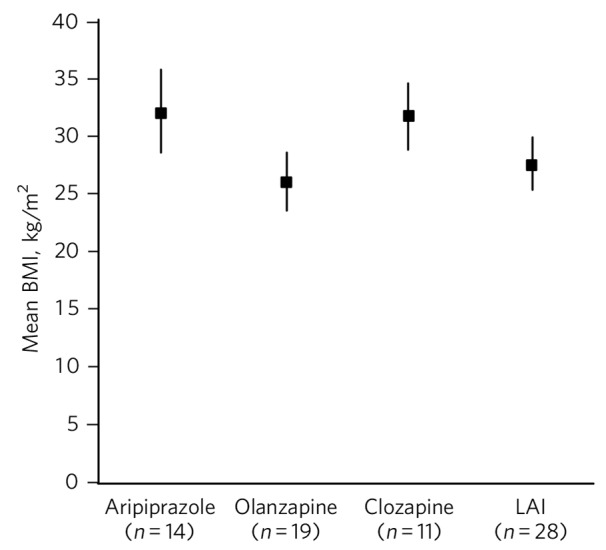
Mean body mass index (BMI) (kg/m^2^) for specific antipsychotics (vertical bars represent 95% confidence intervals). LAI, long-acting injection.

**Fig. 2:**
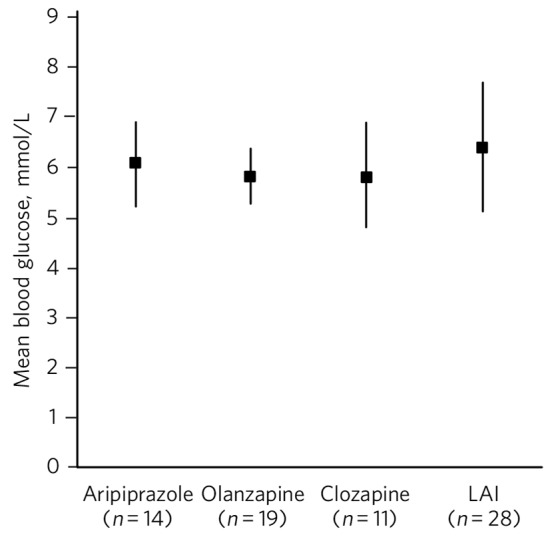
Mean blood glucose levels (mmol/L) for specific antipsychotics (vertical bars represent 95% confidence intervals). LAI, long-acting injection.

Figure 1 gives mean BMIs for various antipsychotics. One-way ANOVA showed a significant difference between the groups (F(3,58) = 3.3, *P* = 0.025) and further analysis showed significant differences between aripiprazole and olanzapine (t(21) = 2.3, *P* = 0.034) and olanzapine and clozapine (t(18) = 2.6, *P* = 0.017). Figure 2 shows mean blood glucose levels for various antipsychotics, but no significant difference between the groups (F(3,54) = 0.17, *P* = 0.91) was observed on one-way ANOVA.

Online Table DS1 describes in further detail the three antipsychotics; in addition to mean dose which was also given in Table 2, median treatment durations with ranges were also recorded. The median duration of treatment was lengthy at 350 days for olanzapine, 735 for clozapine and 315 for aripiprazole. Table DS1 contains previously prescribed medications as these may also have had an influence, particularly in patients who had been on one of the three antipsychotics for a short duration. In the case of aripiprazole, two patients had been on olanzapine for a median duration of 920 days and five patients had been on quetiapine for a median duration of 393 days. The dosage and duration of long-acting antipsychotic injection prior to the initiation of clozapine and aripiprazole was of interest. Patients eventually commencing clozapine were treated with much higher median percentage BNF maximum dosage^9^ allowed than those commencing aripiprazole (62.3% *v*. 6.8%). In addition, patients eventually commencing clozapine were treated for a much longer median duration of long-acting antipsychotic injection (991 *v*. 256 days).

## Discussion

Our primary null hypothesis was that in clinical practice, BMI and blood glucose levels for psychiatric populations would not differ significantly from the local non-psychiatric population, which was supported for BMI. For the psychiatric population several factors may be considered in addition to antipsychotic medication-induced weight gain. Such patients often suffer from lack of motivation as part of their primary mental disorder or possible comorbid mental or substance use disorder. They may be more likely to neglect their physical health,^10^ perform less exercise and make poor dietary choices either by habit or relative expense of some healthy foods including fresh fruit and vegetables. For the general practice population, other factors may be considered including poor dietary choices (high-sugar, high-calorie foods) and convenience of take-away or ready-to-microwave meals.

The Scottish mean BMI in 2012 was 27.3 kg/m^2^ for both men and women. The mean BMI of adults aged 16–64 increased from 25.8 kg/m^2^ in 1995 to 27.1 kg/m^2^ in 2012, with little change since 2008.^11^ Our general practice cohort mean BMI was 2.6 kg/m^2^ higher than the Scottish average. Interestingly, a study from New York found high BMI to be an independent risk factor for re-admission to an in-patient psychiatric unit.^12^ Incidentally, the study's median BMI at 28.5 kg/m^2^ was comparable to our psychiatric populations. In our locality, Lanarkshire, Scotland, 65% of adults are defined as overweight, that is, their BMI is 25 kg/m^2^ or above.^13^ In our severe and enduring mental illness population 81% and in the general practice population 72% had a BMI of 25 kg/m^2^ or above. Being overweight is a known risk factor for cardiovascular disease and diabetes mellitus.^14^

In terms of blood glucose level, our null hypothesis was not supported – blood glucose levels were significantly lower in the general practice population than in the psychiatric population. Again, antipsychotics and other psychotropic medications may be important aetiological factors along with poor dietary choices, with high sugar, fat and salt content in ready or microwave-prepared meals as seen in clinical practice. Schizophrenia and diabetes have been associated comorbid conditions long before antipsychotic medications became available.^15^ A contemporary study of 1642 patients from Australia showed that psychosis itself was associated with insulin resistance as well as high BMI. These findings were seen at a relatively early age, even prior to the start of treatment with antipsychotics^16^ and we support the view of the authors who recommend close monitoring and adherence to guidelines^17^ for all patients commenced on antipsychotics who may be at risk of metabolic syndrome. Our relatively elevated blood glucose measures for the psychiatric populations may suggest insulin resistance which is a precursor of type 2 diabetes mellitus and therefore an additional risk factor for cardiovascular disease.^18^

Our secondary null hypothesis was that in clinical practice, there would be no differences between the three antipsychotics of interest regarding BMI or blood glucose when compared with patients on all other antipsychotics. For blood glucose this null hypothesis was supported. Our blood glucose findings were in keeping with a large systematic review of randomised controlled trials (total *n* = 6239) looking at mostly aripiprazole *v*. olanzapine for the treatment of schizophrenia.^19^ However, for BMI our null hypothesis was not supported. The findings seemed counterintuitive in that patients on aripiprazole had a significantly higher mean BMI compared with other antipsychotics. We considered that prior to aripiprazole, two patients had been on olanzapine and five on quetiapine for a significantly long period of time. This may have contributed to the overall high mean BMI seen in the aripiprazole group. A further consideration is that aripiprazole may have been prescribed specifically for patients who had already gained weight from the effects of a previously prescribed antipsychotic. This phenomenon is known as channelling bias and occurs in observational studies. Observational studies are limited in that causality cannot be inferred. Channelling bias is well recognised and can be mitigated.^20^ However, in our study the lengthy median duration of treatment with aripiprazole at 315 days supports the significant influence of aripiprazole itself in terms of final mean BMI. Furthermore, olanzapine, an antipsychotic known to have a propensity for causing early weight gain, was associated with a statistically significantly lower mean BMI compared with aripiprazole and clozapine. Prior to olanzapine treatment, the antipsychotics prescribed did not give rise to suggestions for why this body mass finding occurred, for example, many patients had been on potentially weight-neutral antipsychotics for a significant period beforehand. The possibility of channelling bias or actively, or perhaps even subconsciously, selecting patients who had a relatively low BMI to start with as being suitable for being commenced on olanzapine may be a consideration. The position that olanzapine is associated with higher BMI and blood glucose levels compared with other antipsychotics was not supported by our findings. This is in keeping with the finding that people who already had a high BMI at the start of treatment may not put on as much weight or that there is a plateau effect at around 39 weeks with long-term treatment with olanzapine.^21^ Our cohort of olanzapine patients had a median duration of treatment of 50 weeks (350 days) suggesting that this plateau period had been reached.

### Limitations

There were some important limitations to this study. There were significant differences in gender distribution and age between the psychiatric and general practice populations. The observational nature of the study does not support suggestions about causality in any direction in terms of BMI, blood glucose or other physiological parameters from particular antipsychotic medications. Randomised controlled trials allow speculation about causality, however, stringent entry requirements for randomised controlled trials exclude many patients seen in clinical practice which our clinical cohorts represent. Due to limitations of data we were unable to report on other important potential metabolic disturbances with the three main drugs of interest, for example, cholesterol, triglycerides and high-density lipoprotein. The sample size of our severe and enduring mental illness group was relatively small (*n* = 46) and consisted mainly of patients with diagnoses of bipolar affective disorder and schizophrenia. Attendance at the physical health monitoring clinic was on a voluntary basis and this may result in a participation bias. This meant that individuals with the most severe debilitating illness and those troubled primarily by negative symptoms, particularly lack of motivation to attend the physical health screening clinic, may have effectively been excluded from our findings. To reliably report our main findings replication with larger psychiatric and general practice populations would be necessary. We would like to underline the importance of further potential confounding effects of other medications which may have an effect on weight gain, for example, lithium, other mood stabilisers and antidepressant medications, antipsychotic polypharmacy (for our psychiatric population *n* = 8), other polypharmacy such as treatment with anti-hypertensive or anti-diabetic medications, and pre-existing medical conditions such as hypothyroidism.

Although BMI recording is now part of routine psychiatric assessment in our locality for both community and in-patients, we were not able to report on BMI or blood glucose at the commencement of antipsychotic medication. We strongly recommend this as part of the careful assessment of patients starting on antipsychotics, particularly as studies show that early rapid weight gain predicts further long-term weight gain and there are effective strategies to counteract this.^22^
